# On Some Urinary Infections, with Especial Reference to Their Treatment by Urotropin
^1^Read before the Bristol Medico-Chirurgical Society, Jan. 8th, 1902.


**Published:** 1902-03

**Authors:** J. Odery Symes

**Affiliations:** Assistant-Physician, Bristol General Hospital


### ON SOME URINARY INFECTIONS, WITH ESPECIAL REFERENCE TO THEIR TREATMENT BY UROTROPIN.[1]

BY

J. ODERY SYMES, M.D., D.P.H.,

*Assistant-Physician, Bristol General Hospital.*

---

THE initial difficulty in investigating any urinary infection is to ensure that the sample of urine is obtained free from any accidental contamination. This may be attempted in one of three ways; namely, by supra-pubic puncture and aspiration; by sterilising the meatus urinarius, directing the patient to pass a small quantity of urine, and then receiving the residue in a sterile bottle; or, after first cleansing the meatus, by drawing off the water by means of a well-boiled rubber catheter, using as a lubricant a solution consisting of hyd. iod. $\frac{3}{10}$ gr., powdered soap ʒxii, glycerine ʒvj., water ʒvj. Sterile urine may be obtained in any of these ways, and may be kept in a warm incubator for weeks without showing signs of bacterial growth. Very frequently, however, with the most stringent precautions, it will be found that after a few days' incubation the urine shows evidences of bacterial growth, and from a number of observations on the urine of healthy persons, I am of opinion that, apart from accidental contamination, micro-organisms may be present in this excretion without there being any departure from normal health. The work of looking for micro-organisms in the urine is much simplified if, instead of examining the whole sample at once, it is placed in the warm incubator for a week. The number of micro-organisms is thus much increased, and cultures may be made on plates or in tubes, and films made from the sediment obtained by centrifugalising in sterile pipettes. The centrifuge I have used has been Purdy's, driven by electricity, and this I have found to be a most satisfactory instrument.

[1] Read before the Bristol Medico-Chirurgical Society, Jan. 8th, 1902.

It is often a matter of extreme difficulty to locate the exact portion of the urinary tract in which the lesion is situated. The rule that pus in acid urine is derived either from the urethra, ureters, or kidney, and pus in alkaline urine from the bladder, is only partly correct; and it is seldom possible, in my experience, to differentiate the various forms of epithelium. To form a correct estimate of the site of the lesion it is necessary to have a history of the clinical progress of the case in order to supplement the chemical and microscopical evidence.

The simplest form of urinary infection is the condition known as *bacilluria*, in which the urine, without necessarily being altered in composition, is loaded with bacteria, which may give it a cloudy appearance. This is most frequently met with during and after the fourth week of enteric fever, and may persist for weeks or months. As typhoid bacilli are never present in the blood in large numbers, but few can be eliminated by the kidney, and multiplication must therefore take place in the bladder. This is no doubt aided by the fact that, owing to the exhausting nature of the disease, the organ is imperfectly or infrequently emptied.

I have examined seven typhoid urines. Of these, one, which was acid and cloudy, showed the B. typhosus in large numbers. This case had been eleven days convalescent. In a second case, in which the urine presented the same appearance and reaction, the organism present was the B. coli; and in a third, in which the urine was alkaline and cloudy, cocci were present. Three were sterile, and in one in which cocci were found in acid urine there had possibly been accidental contamination. Typhoid bacilluria is probably present in not more than ten per cent. of all cases; but as it is certain that a few bacilli are present from time to time, it is advisable to always disinfect the urine.

One other case of bacilluria has come under my notice; namely, an infection by B. coli in a girl during an attack of scarlet fever. Whilst it is possible that bacteria may migrate to the bladder from neighbouring organs, or enter *per urethram*, in the majority of cases the infection takes place by the kidney. That this is the not unusual track is evidenced by the fact that if cultures be taken from the kidneys of persons dying from such acute infectious processes as enteric fever and diphtheria, the specific bacilli are frequently found. Bacilluria sometimes gives rise to blood and pus in small quantities in the urine, but neither of these were found in any of the cases now recorded.

*Tubercle.*—It has recently been shown that in about fifty per cent. of chronic cases of phthisis, tubercle bacilli are present in the urine, although there may be no lesion of any part of the urinary tract. The condition never becomes a true bacilluria, but is sufficient to warrant the disinfection of the urine, a procedure which at the present time is certainly not practised. Although the introduction of the high-speed centrifuge and specially adapted sedimentation tubes has done much to facilitate the detection of the tubercle bacilli in urine, there yet remains a considerable margin for error, and negative results cannot be accepted as final until inoculation experiments have been made. It is much to be regretted that no facilities exist for such a test at present in Bristol. I have found tubercle bacilli in four out of eleven specimens of urine from suspected cases of tubercular infection. Two of these were cases of pyelonephritis, one of pyelitis, and the fourth was diagnosed as a case of tubercular ulceration of the bladder.

In acute pyelitis and pyelonephritis (non-tubercular), the urine is often alkaline. This may partly be due to the admixture of blood. One such case comes into the present series; namely, that of a soldier who had been discharged from the service shortly after an attack of fever, apparently of a septic nature. He developed acute pyelonephritis whilst under observation, the infecting organisms being staphylo- and streptococci. There were at first a few casts in the urine, but latterly these could not be detected, and the disorder lapsed into the chronic stage. Another case of acute pyelonephritis, confirmed by *post-mortem* examination, was that of an old man, a diabetic, who died of pyæmia following cellulitis. The infecting organism was the staphylococcus aureus. These cases illustrate the possibility of infecting the pelvis of the kidney and the lower urinary tract from the kidney and general circulation; it is more common, however, for infection to spread from below, ascending from the bladder. In some cases it is difficult to say where the infection has started, as in the case of a man with urinary obstruction due to pressure of a malignant growth on the urethra. This patient had slight cystitis and pyelitis, which improved under treatment; but a *post-mortem* examination, some months later, showed a ureter distended with pus and a kidney riddled with abscesses. The invading organism was a streptococcus. No instrument had been passed *per urethram*, and at the time the urine was examined there was not any ulceration into the genito-urinary tract. The bacillus coli is an organism often associated with pyelitis, but was not found in the present series. One chronic case of pyelonephritis was due to a diplococcus.

*Cystitis.*—Seven cases of cystitis were investigated. Of these the urine was acid in three, and alkaline in four. Of the three cases with acid urine, the infecting organism was in two the bacillus coli, and in the third a streptococcus. One of the coli infections was in a man, and the organism had probably been introduced by catheterisation; the second case was in a woman, and for this attack no cause could be assigned. The bacillus typhosus and B. lactis ærogenes may also excite acid cystitis.

The four cases of alkaline cystitis were all due to mixed infections. Two were of post-gonorrhœal origin. In one of these staphylococci and (?) gonococci were found, and in the second staphylococci and bacilli. Of the remaining cases, one showed diplococci, bacilli, and spirilla; and the other staphylococci and B. coli. Alkaline cystitis is almost invariably due to the presence of staphylococci, but may also be excited by the proteus bacillus.

With regard to the special treatment of these infections by urotropin, there are two theories brought forward to explain the therapeutic action of this drug in urinary infections. Nicolaier maintains that at the body temperature, and in the presence of uric acid and acid salts, urotropin becomes decomposed in the urine with the formation of formaldehyde. That such a decomposition occurs at higher temperatures, and in the presence of free acid, there is no doubt; but many observations go to show that no such change takes place at so low a temperature as 37·5° C. Cammidge has brought forward evidence to prove that urotropin forms with acid urine, in the kidney, a compound body which possesses antiseptic properties, and it is to this body (the nature of which is not clear) that its therapeutic action is due. Simple watery solutions of urotropin have some inhibitory and germicidal action ; but, although urotropin is in part excreted unchanged in the urine, the bactericidal action cannot be due to the unchanged drug, for it is only by using *very* strong watery solutions that even its inhibitory power can be demonstrated, and it is never possible to bring the urine up to this standard of concentration.

I have tested the inhibitory and germicidal powers of urotropin with cultures of staphylococci, B. coli, and B. typhosus. The cultures were inoculated into, or mixed with, acid urine, to which urotropin had been added so as to make a solution of the strength of 1 in 1000, and then kept in the incubator at 37° C. The inhibitory power was marked in all cases. The bactericidal power, however, varied, being much more marked in the case of the B. typhosus than in the case of the others. The B. lactis ærogenes, which often occurs in cases of cystitis, rapidly dies out in urines to which urotropin is added. Streptococci are more resistant.

The germicidal action is more pronounced if the infected urine be kept in the incubator at 37.5° C. than if left at the room temperature. It also apparently loses much of its power if the urine be originally alkaline. This I was able to demonstrate by using the urine from a case of phosphaturia.

These variations in the behaviour of the drug towards different organisms and conditions of medium and temperature, in part explain the varying results obtained with it clinically.

In bacilluria it has a speedy and permanent curative action, because here the micro-organisms are swimming freely in the urine, which is acid when excreted, and remains so throughout the duration of the disease.

In pyelitis and pyelonephritis, on the other hand, although urotropin generally relieved the symptoms, in none of the cases, tubercular or non-tubercular, did a cure result. The chief reason for this undoubtedly is, that in these cases the invading organisms are deeply imbedded in the tissues, and so cannot be reached by the drug. Another possible reason may be that in acute pyelitis and pyelonephritis, the urine is sometimes alkaline, and the complex antiseptic body, which Cammidge describes as only being formed with acid urine, is consequently not present.

The best results with urotropin are seen in cases of cystitis secondary to enlargement of the prostate, probably because in such cases the urine on leaving the kidney is acid, and the bladder walls are not deeply penetrated by bacteria. The antiseptic body formed by the urotropin first inhibits the growth of and then kills the bacteria, which give rise to the alkalinity of the urine ; and the bladder, freed from this source of irritation, will, if the drug be resumed from time to time, remain permanently in good working order. Presumably cystitis secondary to stricture of the urethra would give equally favourable results.

Cases of cystitis secondary to gonorrhœa do not, in my experience, do well on urotropin. The symptoms are relieved, and the patient continues to take the drug because of the relief; but in two cases under my care, in both of which the urine was originally alkaline, it was possible to detect organisms in the acid urine after the drug had been taken more or less steadily for in one case five, and the other six, months. Cessation from urotropin in such cases generally means a return of the symptoms.

Cystitis due to B. coli, accompanied with acid urine, such as is seen so frequently in women, does not yield readily to urotropin, probably because this organism is peculiarly resistant to the drug. I have found freely motile bacilli in the urine of a patient, suffering from vesical coli infection, who, for twelve days previously, had been taking ten grains of urotropin three times a day.

My custom has been to give urotropin in doses of ten grains four times daily, the last dose on going to bed at night. In none of the cases reported has hæmaturia been noted.

